# Yttrium-90-Labeled Anti-Glypican 3 Radioimmunotherapy Halts Tumor Growth in an Orthotopic Xenograft Model of Hepatocellular Carcinoma

**DOI:** 10.1155/2019/4564707

**Published:** 2019-09-15

**Authors:** Andrew D. Ludwig, Kevin P. Labadie, Y. David Seo, Donald K. Hamlin, Holly M. Nguyen, Vimukta M. Mahadev, Raymond S. Yeung, D. S. Wilbur, James O. Park

**Affiliations:** ^1^Department of Surgery, University of Washington, Seattle, WA, USA; ^2^Department of Radiation Oncology, University of Washington, Seattle, WA, USA; ^3^Department of Urology, University of Washington, Seattle, WA, USA

## Abstract

Hepatocellular carcinoma (HCC) is the second most lethal malignancy globally and is increasing in incidence in the United States. Unfortunately, there are few effective systemic treatment options, particularly for disseminated disease. Glypican-3 (GPC3) is a proteoglycan cell surface receptor overexpressed in most HCCs and provides a unique target for molecular therapies. We have previously demonstrated that PET imaging using a ^89^Zr-conjugated monoclonal anti-GPC3 antibody (*α*GPC3) can bind to minute tumors and allow imaging with high sensitivity and specificity in an orthotopic xenograft mouse model of HCC and that serum alpha-fetoprotein (AFP) levels are highly correlated with tumor size in this model. In the present study, we conjugated ^90^Y, a high-energy beta-particle-emitting radionuclide, to our *α*GPC3 antibody to develop a novel antibody-directed radiotherapeutic approach for HCC. Luciferase-expressing HepG2 human hepatoblastoma cells were orthotopically implanted in the livers of athymic nude mice, and tumor establishment was verified at 6 weeks after implantation by bioluminescent imaging and serum AFP concentration. Tumor burden by bioluminescence and serum AFP concentration was highly correlated in our model. Yttrium-90 was conjugated to *α*GPC3 using the chelating agent 1,4,7,10-tetraazacyclododecane-1,4,7,10-tetraacetic acid (DOTA) and injected via the tail vein into the experimental mice at a dose of 200 *μ*Ci/mouse or 300 *μ*Ci/mouse. Control mice received DOTA-*α*GPC3 without radionuclide. At 30 days after a single dose of the radioimmunotherapy agent, mean serum AFP levels in control animals increased dramatically, while animals treated with 200 *μ*Ci only experienced a minor increase, indicating cessation of tumor growth, and animals treated with 300 *μ*Ci experienced a reduction in serum AFP concentration, indicating tumor shrinkage. Mean tumor-bearing liver weight in control animals was also significantly greater than that in animals that received either dose of ^90^Y-*α*GPC3. These results were achieved without significant toxicity as measured by body condition scoring and body weight. The results of this preclinical pilot demonstrate that GPC3 can be used as a target for radioimmunotherapy in an orthotopic mouse model of HCC and may be a target of clinical significance, particularly for disseminated HCC.

## 1. Introduction

Hepatocellular carcinoma (HCC) is recognized as the fifth most common cancer and second leading cause of cancer-related deaths worldwide, resulting in over 750,000 deaths annually [1]. Though there has been progress in surgical and nonsurgical treatment for HCC, the prognosis remains poor, particularly for late-stage disease. Sorafenib, a multikinase inhibitor and the best form of chemotherapy for advanced disease, has been shown to prolong median survival and time to progression by only 3 months [2]. Because of this dismal prognosis, novel targets and therapies are desperately needed.

Glypican-3 (GPC3) is a heparan sulfate proteoglycan found on the cell surface of human embryonic stem cells. It is anchored by glycosylphosphatidylinositol and regulates growth and morphogenesis through insulin-like growth factor and hedgehog signaling pathways [3, 4]. Its importance in regulating cell growth is underscored by a loss-of-function mutation in GPC3 that causes Simpson–Golabi–Behmel syndrome, a condition of skeletal and organ overgrowth [5]. Expression of GPC3 in the fetal liver is observed from 18 to 30 weeks of gestation, but no GPC3 expression is seen in normal adult liver cells [3, 6, 7]. Conversely, high expression of GPC3 is seen in HCC, is correlated with AFP expression, and can be used to differentiate HCCs from benign liver lesions [8–10]. In addition, the level of expression of GPC3 in HCC patients is correlated with poorer prognosis and risk of recurrence after primary resection or liver transplant [11–15].

Because of this differential expression and cell surface location, GPC3 is a promising tumor marker for diagnostic and therapeutic purposes in HCC. We have previously demonstrated the utility of antibody-directed ^89^Zr radioisotopes as PET contrast agents to identify GPC3-expressing orthotopic liver tumors *in vivo* [16, 17]. In the current report, we describe the development of a novel antibody-directed therapeutic radioisotope in the form of a ^90^Y-*α*GPC3 conjugate and study its effect on *in vivo* HCC tumor growth in our orthotopic mouse model.

## 2. Materials and Methods

### 2.1. Cell Lines and Tissue Culture

Luciferase-expressing GPC3-positive HepG2-Red-FLuc HCC cells were purchased from PerkinElmer (Bioware, cat. no. BW134280). Cell lines were maintained in a monolayer at 37°C in Dulbecco's modified Eagle's medium (DMEM; Gibco) supplemented with 10% fetal bovine serum (FBS; Gibco) in a humidified atmosphere of 95%/5% air/CO_2_.

### 2.2. Anti-GPC3 IgG1 Generation (as Previously Described [16])

RBF/DnJ mice were immunized with recombinant carrier-free human GPC3 protein in Freund's adjuvant solution. After several boost injections, antiserum ELISAs confirmed the presence of the *α*GPC3 IgG. Additional boost injections were delivered to ensure IgM/IgG switch, which was verified on ELISA with IgG titrated to 1 : 10,000. After final prefusion boost injections, mice were euthanized, their spleens were harvested, 1 × 10^8^ splenocytes were fused on a ratio of 1 : 1 with FOX-NY myeloma cells, and the resultant hybridomas were resuspended in adenine/aminopterin/thymidine FBS solution. Clones producing high titers of GPC3 IgG1 were selected using capture ELISA with goat antimouse IgG1 for isotyping.

### 2.3. Production of ^90^Y-*α*GPC3 Antibody

To demetallate the *α*GPC3 antibody, it was dialyzed against metal-free HEPES (50 mM HEPES (*N*-(2-hydroxyethyl)piperazine-*N*′-ethanesulfonic acid), 150 mM NaCl, and 1 mM EDTA adjusted to 8.5 pH and passed over a Chelex 100 (Biorad) column to remove metals) with a minimum of 6 buffer changes over 3 days at 4°C using Chelex 100 resin at each buffer change to scavenge metals. The metal-free *α*GPC3 was added to a DOTA-Bn-NCS (Macrocyclics) solution (10 mg/mL in DMSO), and the reaction was allowed to run overnight at room temperature with gentle mixing. The reaction mixture was then dialyzed against a metal-free citrate buffer (50 mM sodium citrate and 150 mM NaCl with pH 5.5) over 3 days at 4°C followed by dialysis against 150 mM saline for another 3 days. Each buffer change contained Chelex resin to scavenge metals. Demetallated ammonium acetate (500 mM, pH 5.3) and ^90^Y were combined followed by *α*GPC3-DOTA, prepared as above, and incubated at 45°C for 1 hr before cooling to room temperature. The reaction was quenched with diethylenetriamine pentaacetic acid (DTPA). The labeled antibody was then separated from unreacted ^90^Y via a PD-10 column (GE Healthcare) and eluted in PBS prior to analysis by thin layer chromatography (TLC) to verify radiochemical purity. Acid-washed vials and pipette tips were used for all steps.

### 2.4. Flow Cytometry

In vitro binding of the DOTA-*α*GPC3 conjugate was evaluated by flow cytometry. HepG2-Red-FLuc cells were grown as above until 70% confluent and then detached with 0.25% trypsin, counted, washed, and resuspended in cold phosphate-buffered saline (PBS) at a concentration of 1 × 10^6^ cells/mL. One microgram of unconjugated or DOTA-*α*GPC3 primary antibody was added to the cell suspension and incubated for 45 minutes on ice. Primary antibody control samples received 1 *μ*g of isotype-matched IgG1 control antibody (BD Biosciences, cat. no. 555746). Unstained samples did not receive primary antibody. The cells were then washed in cold PBS, and 1 *μ*g of FITC-labeled goat-*α*-mouse IgG1 secondary antibody (Southern Biotech, cat. no. 1070-02) was added to the cell suspension and incubated on ice for 30 minutes in the dark. Unstained samples did not receive secondary antibody. The cells were washed in cold PBS, fixed with 1% paraformaldehyde for 15 minutes, and then washed and resuspended in cold PBS. Fixed cells were analyzed with a BD FACSCanto flow cytometer (Becton Dickinson Biosciences, Franklin Lakes, NJ) using the FACSDiva software. A minimum of 10,000 cells were analyzed for each sample in triplicate. Data analysis was performed on the FlowJo software, version 8.8.6 (Tree Star, Ashland, OR).

### 2.5. Animal Models

All animal studies were performed in accordance with the University of Washington Office of Animal Welfare guidelines for the humane use of animals, and all procedures were reviewed and approved by the Institutional Animal Care and Use Committee. To generate the orthotopic xenograft model, 8-week-old female athymic Nu/J mice (The Jackson Laboratory) were anesthetized using 1.5% inhaled isoflurane, and the left lobe of the liver was exposed through an upper midline laparotomy. HepG2-Red-FLuc cells (2 × 10^6^) in 50 *μ*L of Dulbecco's modified Eagle's medium containing 50% Matrigel (BD Biosciences) were injected into the subcapsular space of the left lobe.

### 2.6. Bioluminescent Imaging

Six weeks after orthotopic HepG2 cell injection, a 75 mg/kg intraperitoneal injection of VivoGlo luciferin (Promega) was administered and imaging was performed using an IVIS Lumina II system (PerkinElmer) to verify tumor establishment and monitor the growth of intrahepatic tumors. Tumor size was calculated based on the average photon emission (photons/sec) in a 2D region of interest (ROI) covering the entire animal. The ROI was corrected for background bioluminescence, and the size of the ROI (32 cm^2^) was identical for every animal imaged. Calculations were performed using the Living Image software (version 4.2; Caliper Life Sciences).

### 2.7. Measurement of Mouse Serum AFP

At the specified times, whole blood was obtained from animals using submandibular bleeding [18] and collected in EDTA-coated Eppendorf tubes. Serum was extracted from the fresh whole blood and then frozen and allowed to decay 10 half-lives (∼27 days) in accordance with the University of Washington Environmental Health and Safety policy. The serum concentration of AFP was determined on the UniCel Dxl 800 Access Immunoassay System (Beckman Coulter) using an Access AFP alpha-fetoprotein pack (Quest Diagnostics).

### 2.8. *In Vivo* Radioimmunotherapy

Animals bearing established tumors as determined by IVIS imaging and serum AFP concentration using the above methods were randomly assigned to three experimental groups. All animals received the antibody conjugate via tail vein injections. Control animals were injected with 70 *μ*g DOTA-conjugated *α*GPC3 antibody without radioisotope. Treated animals received 70 *μ*g ^90^Y-*α*GPC3 at the specified dosages of radionuclide. At 14 days after antibody injection, serum AFP was measured based on the above protocol. At 30 days after antibody injection, the animals were euthanized, blood was extracted via cardiac puncture, and livers were harvested, wet-weighed, and then placed in 10% (w/v) neutral-buffered formalin.

### 2.9. Statistical Analysis

All numeric data are expressed as mean ± SEM unless otherwise indicated. Excel (version 12.0.6; Microsoft) was used for statistical analysis. For continuous variables, an unpaired, 2-tailed Student's *t*-test was used. For multigroup comparisons, a one-way analysis of variance (ANOVA) was used. In all cases, a *p* value ≤0.05 was considered statistically significant.

## 3. Results

### 3.1. Flow Cytometry

**Figure 1:**
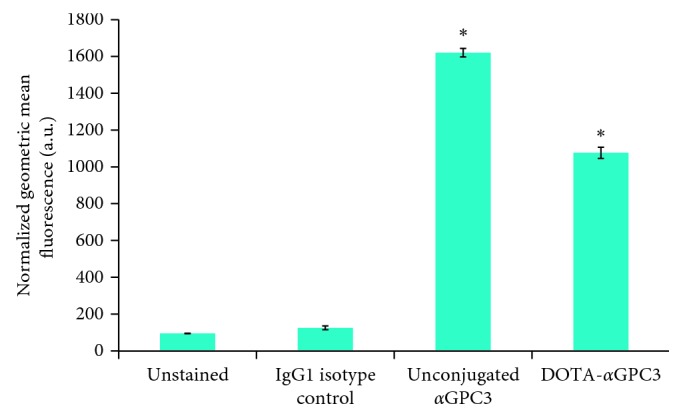
DOTA conjugation maintains *α*GPC3 binding *in vitro.* Flow cytometry on fixed HepG2 cells demonstrates a significant increase in mean fluorescence of both unconjugated and DOTA-conjugated *α*GPC3 compared to unstained cells and isotype-matched primary antibody control samples (^*∗*^*p* < 0.001). Error bars represent standard deviation of triplicate measurements for >10,000 cell counts per sample.

Flow cytometry confirmed binding of both unconjugated and DOTA-conjugated *α*GPC3 to the luciferase-expressing HepG2-Red-FLuc cell line (Figure 1). The normalized geometric mean fluorescence of both unconjugated (1619.7 ± 23.2 a.u.) and DOTA-conjugated *α*GPC3 (1076 ± 30.0 a.u.) is significantly greater than that of unstained (95.2 ± 1.4 a.u.) and IgG1 isotype-matched primary antibody control (125.7 ± 10.5 a.u.) samples (*p* < 0.001 vs. isotype control). Though DOTA conjugation did appear to marginally affect *α*GPC3 antibody binding *in vitro*, mean fluorescence of conjugated samples remained >8-fold higher than that of control samples.

### 3.2. Orthotopic Tumor Establishment

**Figure 2:**
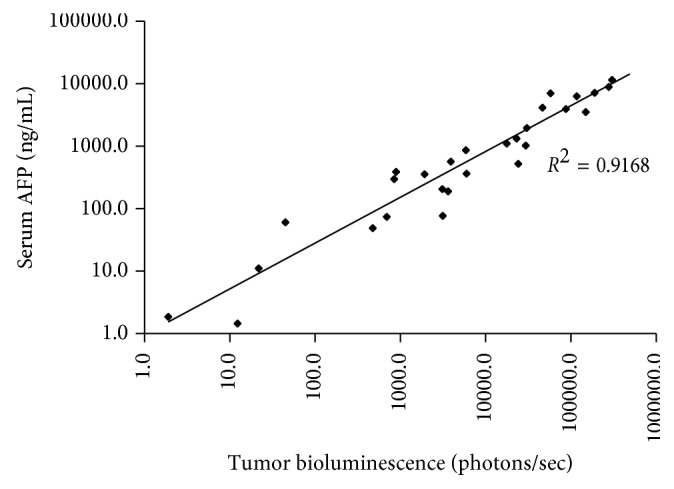
Plot of serum AFP concentration compared to tumor bioluminescence by IVIS imaging in tumor-bearing mice. Serum AFP concentration in mice 6 weeks after orthotopic HepG2-Red-FLuc xenograft implantation is highly correlated with tumor bioluminescence by IVIS imaging, indicating that AFP excretion by HepG2 orthotopic xenografts is dependent on tumor size.

Serum AFP concentration was measured in tumor-bearing mice at six weeks after HepG2-Red-FLuc orthotopic implantation. Tumor establishment was verified by IVIS imaging at the time of serum sampling. Serum AFP levels in study animals ranged from 474.3 ng/mL to 421,600 ng/mL. Tumor bioluminescence ranged from 48.6 photons/sec to 50,340 photons/sec. Tumor bioluminescence as demonstrated by IVIS imaging correlates with serum AFP concentration with a correlation coefficient *R*^2^ of 0.92 (Figure 2). This finding indicates that serum AFP concentration can be used to monitor tumor growth and response to treatment.

### 3.3. Radioimmunotherapy

**Figure 3:**
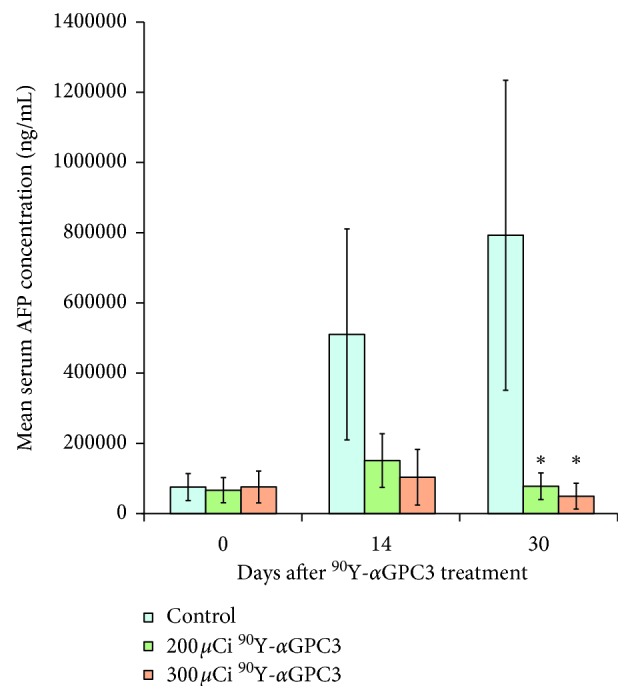
Mean serum AFP concentration of tumor-bearing mice at specified time points after administration of radioimmunotherapy. Serum AFP steadily increases over time in control animals (*n* = 7), while AFP concentration in animals treated with 200 *μ*Ci (*n* = 9) or 300 *μ*Ci (*n* = 9) of the ^90^Y-*α*GPC3 conjugate remained at pretreatment levels and was significantly lower than that in control animals by 30 days (^*∗*^*p* < 0.05). Error bars represent standard error of the mean concentration.

To track tumor growth and response to radioimmunotherapy treatment, serum AFP concentration was monitored at 0, 14, and 30 days after administration of either low-dose (200 *μ*Ci, *n* = 9) or high-dose (300 *μ*Ci, *n* = 9) ^90^Y conjugated to 70 *μ*g of DOTA-*α*GPC3 via tail vein injection. Control animals (*n* = 7) received DOTA-*α*GPC3 without radionuclide. At the time of antibody injection, mean serum AFP concentration for control (75,258 ± 38,683 ng/mL), low-dose (66,434 ± 35,895 ng/ml), and high-dose (75,568 ± 45,467 ng/mL) groups was not statistically significantly different (*p*=0.98), indicating equivalent overall tumor burden between groups at the initiation of the therapy (Figure 3).

At 14 days after antibody injection, mean serum AFP concentration of control animals increased by 578% (to 510,284 ± 300,473 ng/ml), whereas animals that received low-dose radioimmunotherapy treatment experienced a 127% increase (to 150,800 ± 76,392 ng/mL) and animals that received high-dose treatment only experienced a 37% increase (to 103,344 ± 79,120 ng/mL). Though this trend was strong, the mean change in serum AFP concentration at 14 days between control and low-dose or high-dose treatment groups did not reach statistical significance (*p*=0.16 and *p*=0.10, respectively). The mean serum AFP concentration of animals treated with low-dose and high-dose ^90^Y-*α*GPC3 was not statistically different from each other at 14 days (*p*=0.67) and was not statistically different from pretreatment levels (*p*=0.33 and *p*=0.76, respectively).

By 30 days after radioimmunotherapy administration, mean serum AFP concentration of control animals had increased by 953% (to 792,566 ± 441,503 ng/mL) from pretreatment levels, while animals that received low-dose radioimmunotherapy treatment experienced a modest 17% increase over 30 days (to 77,820 ± 37,895 ng/mL) and animals that received high-dose treatment saw a decrease in mean serum AFP concentration by 35% (to 49,342 ± 36,800 ng/mL).

Notably, both low-dose and high-dose treatment groups experienced a decrease in mean serum AFP concentration from 14 days to 30 days by 48% and 52%, respectively. The mean change in AFP concentration over the 30-day study period was significantly different between control and low-dose groups, between control and high-dose groups, and between low- and high-dose groups (*p* ≤ 0.05). However, the mean serum AFP concentrations of low-dose and high-dose treatment groups did not differ statistically from each other at 30 days after antibody injection (*p*=0.60).

**Figure 4:**
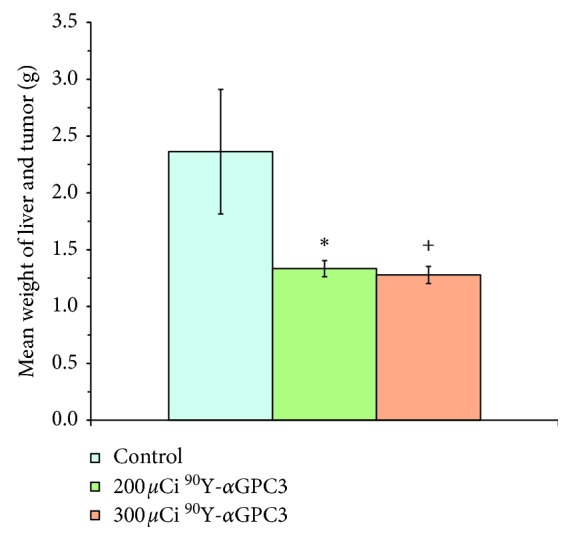
Mean weight of tumor-bearing livers at the conclusion of the 30-day radioimmunotherapy trial. The weight of tumor-bearing livers resected en bloc is increased in control animals compared to those treated with either 200 *μ*Ci or 300 *μ*Ci ^90^Y-*α*GPC3 conjugate (^*∗*^*p*=0.05; ^+^*p* < 0.05). Error bars represent standard error of the mean organ weight.

At 30 days after radioimmunotherapy treatment, study animals were euthanized and livers were removed en bloc to gauge tumor growth. The mean weight of control animal livers (2.36 ± 0.55 g) was significantly greater than that of animals that received either low-dose (1.33 ± 0.07 g) or high-dose (1.28 ± 0.08 g) ^90^Y-*α*GPC3 treatment (*p* ≤ 0.05; Figure 4). The mean weight of livers from animals receiving low-dose or high-dose ^90^Y-*α*GPC3 treatment did not differ statistically (*p*=0.60). During the course of the study, no animals were noted to have a significant change in body weight or body condition scoring, nor were any animal euthanized prior to the end date of the study.

## 4. Discussion

HCC is a common and deadly form of cancer for which few treatment options exist for late-stage or disseminated disease. In this preclinical study, we report the ability of a novel radioimmunotherapy agent, combining the radionuclide ^90^Y with an HCC-specific antibody targeting the cell surface proteoglycan GPC3, to halt tumor growth in an orthotopic xenograft model.

To create a radioimmunotherapy agent, we conjugated a high-energy beta-emitting radionuclide, ^90^Y, to a tumor-specific antibody using the chelating agent 1,4,7,10-tetraazacyclododecane-1,4,7,10-tetraacetic acid (DOTA). We demonstrated that conjugating this agent to *α*GPC3 maintained binding of the antibody *in vitro* by flow cytometry. We have previously shown that conjugating ^89^Zr to *α*GPC3 with deferoxamine preserves antibody binding and specificity *in vitro* and *in vivo* [16]. Conjugation of ^90^Y to monoclonal antibodies with DOTA is clinically relevant and has been used in clinical trials for the treatment of pancreatic cancer [19], B-cell lymphoma [20], and leukemia [21]. Similarly, ^90^Y itself is used clinically in the treatment of HCC. Radioembolization with ^90^Y has been used in the treatment of HCC since the 1960s [22], and ^90^Y microspheres have been shown to significantly prolong time to progression in HCC when compared to chemoembolization [23]. These efforts have demonstrated that ^90^Y treatment is well tolerated in advanced-stage HCC [24].

In our orthotopic mouse model, we used serum AFP concentration as a corollary of tumor size, allowing a simple blood draw to track the response to treatment while animals were under radionuclide treatment. Serum AFP concentration prior to treatment was highly correlated with tumor size as established by bioluminescent imaging of the luciferase-expressing orthotopic tumors. Because of the intra-abdominal location of these tumors, external measurement of tumor volume is not possible. Others have demonstrated that serum AFP concentration is correlated with tumor size by bioluminescent and magnetic resonance imaging in a HepG2 orthotopic xenograft model [25]. Similarly, this method of correlating bioluminescence with tumor serum markers to track treatment response in orthotopic xenografts undergoing immunotherapy has been utilized successfully in several other tumor models [26]. In the clinical setting, high serum AFP concentration (>400 ng/mL) is considered diagnostic for HCC in the appropriate patient (e.g., cirrhotic or high-risk for HCC) and can be used to monitor the treatment effect and recurrence in patients whose tumors expressed AFP at diagnosis [27]. Serum AFP concentration decreasing in response to therapy in HCC was first described in the late 1970s [28] and continues to be clinically relevant as a predictor of outcome across the spectrum of HCC therapies. A similar phenomenon is present in our orthotopic model of HCC undergoing radioimmunotherapy.

We have previously demonstrated that ^89^Zr-*α*GPC3 can be used as a novel imaging agent in this orthotopic xenograft HCC model. Using the same antibody and similar conjugation techniques, we extend the utility of this antibody to a therapeutic platform using ^90^Y. Naturally, this presents the opportunity to combine tumor-specific diagnostic and therapeutic modalities using the same antibody and introduces the possibility of targeting small tumors and disseminated disease. Studies of this theranostics platform in our orthotopic xenograft model are in process. It is possible that the presence of ^90^Y alone could account for some of the treatment effects seen in our model. However, our study utilized a single dose of radioimmunotherapy, and it is unlikely that such a dose administered systemically without tumor-specific activity could result in the sustained arrest of tumor growth over 30 days seen here. Nevertheless, we anticipate using nontargeting antibody controls in future preclinical experiments.

In addition, the direct cytotoxic effect of *α*GPC3 monoclonal antibodies is widely recognized. Since its characterization as a diagnostic and prognostic marker in HCC, GPC3 has been studied as a therapeutic target. A cytotoxic humanized *α*GPC3 monoclonal antibody, GC33, is capable of inhibiting orthotopic xenograft tumor growth [29], and clinical trials have been underway in HCC patients since 2013 [30]. Though our antibody was not designed with the intention of inducing a cytotoxic effect, control animals received DOTA-conjugated *α*GPC3 to account for the possible direct effect of the antibody itself. Progression of tumor growth in control animals during our study suggests that such an effect, if present, is not significant with our *α*GPC3 antibody.

The tumor-targeting effect of the *α*GPC3 antibody localizes the treatment effect of ^90^Y to the tumor, prolonging the exposure of tumor cells to *β*-particle emission and limiting systemic effects. In the present study, all animals were noted to tolerate radioimmunotherapy without significant morbidity or mortality. Efforts to better quantify potential off-target effects of this novel radioimmunotherapy agent deserve further study and are currently underway.

## 5. Conclusions

In this study, we report the ability of a novel ^90^Y-*α*GPC3 conjugate radioimmunotherapy to successfully halt the growth of luciferase-expressing HepG2 tumors in an orthoptic xenograft model of hepatocellular carcinoma. We used serum AFP as a marker of tumor size, validated by bioluminescent imaging. In our model, serum AFP levels in animals treated with a single dose of 200 *μ*Ci remained at pretreatment levels while in control animals serum AFP increased by over 900% by 30 days after treatment. In animals treated with 300 *μ*Ci of our conjugate, serum AFP levels decreased below pretreatment levels, indicating a reduction in tumor size.

## Data Availability

The data used to support the findings of this study, including values behind means and standard deviations and images of *in vivo* bioluminescence, are available from the corresponding author upon request.
